# Books and Pamphlets

**Published:** 1874-09

**Authors:** 


### BOOKS AND PAMPHLETS.

Clinical Electro-Therapeutics, Medical and Surgical: A Hand-Book for Physicians in the Treatment of Nervous and other Diseases. By Allen McLane Hamilton, M.D., Physician-in-Charge of the New York State Hospital for Diseases of the Nervous System, etc., etc., with numerous Illustrations. New York: D. Appleton & Co.

This is a readable work—one we recommend to the attention of the practitioner upon the very important subjects of which it treats. It covers the ground of electro-therapeutics, in its various modes of application, and to a very numerous class of diseases, fully up to the latest investigations by the most experienced workers in this field of scientific research. Should our readers desire to know something more (and better) of electro-therapeutics, we commend this book to their attention as containing the very "pith" of the subject in the most concise and attractive form, as well as handsomely illustrated.

Insanity in its Relations to Crime: A Text and a Commentary. By William A. Hammond, M.D., Professor of Diseases of the Mind and Nervous System, and of Clinical Medicine, in the Bellevue Hospital Medical College, etc., etc. New York: D. Appleton & Co. 1873.

The "text" for Dr. Hammond's "commentary" is taken from several remarkable subjects of insanity, the history of whose insane manifestations are given at length—subjects committing crime impelled by insane impulse. The commentary is just such as Dr. Hammond alone could give—full of argument, research and illustration. It reads like a romance, while the master-mind of a great thinker sparkles on every page. It is a work every lawyer and every doctor should read; aye, more—it is a work that should be read by intelligent and thinking men in all stations of life.

Transactions of the Medical Society of the State of West Virginia.

This is a well-filled volume of 635 pages. The papers are of considerable merit, and quite numerous. We are indebted to Dr. J. C. Hopp, Treasurer of the Society, for the volume, for which we thank him.

Transactions of the Medical and Chirogical Faculty of the State of Maryland: Seventy-fifth Annual Session, held at Baltimore, Maryland, April, 1874.

A very well-filled volume. A few of the papers, only, are of much practical value.

The Relations of Medical Societies to Progress in Science: Inaugural Address of the President of the Medical Society of the county of Kings, New York—Alex. J. C. Skene, M.D.

Clinical Report of the Lying-in Service at Bellevue Hospital, for the year 1873. By William T. Tusk, M.D.

Writer's Cramp, or Scrivener's Palsy. By Reuben A. Vance, M.D., New York.

The Advantages of Torsion as a Means of Arresting Hemorrhage: Read before the Dallas City Medical Society, Dallas, Texas. By E. T. Easley, A.M,, M.D.

A very creditable monograph of the subject, and one, too, our Texas friends should feel gratified to own as coming from a Texas brother.

Resume of Cataract Operations. By A. W. Calhoun, M.D., Atlanta, Georgia.

Our friend, Dr. Calhoun, has made a fine reputation in the specialties of the diseases of the eye and ear. The pamphlet before us will not detract from, but rather add to, the professional standing of this young Georgian in his chosen field of surgery. We wish him a successful and prosperous career which the future holds out, if we may judge from the laurels won by him in the past.

Herpes Gestatiores: A rare Affection of the Skin, peculiar to Pregnancy. By Duncan Bulkley, M.D., New York.

Address of Joseph M. Toner, M.D., President of the American Medical Association.

Dictionary of Elevations and Climatic Register of the United States, containing, in addition to elevations, the latitude, mean annual temperature, and the total annual rain-fall of many localities, with a brief introduction on the orographic and other physical peculiarities of North America. By J. M. Toner, M.D. A very valuable and useful work.

Syphilitic Membranous Occlusion of the Reina Glottidis. By Louis Elsberg, M.D.

Ophthalmic Contributions: I. Dermoid Tumor of Cornea; II. An additional Method for the Determination of Astigmatism; III. Cysts of the Iris—removal by Operation. By Geo. Strawbridge, M.D., Philadelphia.

Urethrotomy, External and Internal Combined, in Cases of Multiple and Difficult Stricture: with remarks on the Urethral Calibre. By Fressenden N. Otis, M.D., New York. G.

